# Contribution of *BRCA1* germ-line mutations to breast cancer in Greece: a hospital-based study of 987 unselected breast cancer cases

**DOI:** 10.1038/sj.bjc.6605115

**Published:** 2009-06-02

**Authors:** S Armaou, M Pertesi, F Fostira, G Thodi, P S Athanasopoulos, S Kamakari, A Athanasiou, H Gogas, D Yannoukakos, G Fountzilas, I Konstantopoulou

**Affiliations:** 1 Molecular Diagnostics Laboratory, I/R-RP, National Center for Scientific Research ‘Demokritos’, Aghia Paraskevi, 15310 Athens, Greece; 2 BioGenomica, Center for Genetic Research and Analysis, Papanikoli 4, 15232 Athens, Greece; 3 Department of Medical Oncology A, Metaxa Cancer Hospital, 18537 Piraeus, Greece; 4 First Department of Internal Medicine, University of Athens, Laiko Hospital, 11527 Athens, Greece; 5 Department of Medical Oncology, Papageorgiou Hospital, Aristotle University of Thessaloniki School of Medicine, 56429 Thessaloniki, Greece; 6 Hellenic Cooperative Oncology Group, Hadjikonstanti 18, 11524 Athens, Greece

**Keywords:** *BRCA1*, *BRCA2*, hereditary breast-ovarian cancer, genetic screening, germ-line mutation, founder mutation

## Abstract

**Background:**

In most Western populations, 5–10% of all breast cancer cases can be attributed to major genetic factors such as predisposing mutations in *BRCA1* and *BRCA2*, with early-onset cases generally considered as an indicator of genetic susceptibility. Specific *BRCA1* and *BRCA2* mutations or different mutation frequencies have been identified in specific populations and ethnic groups. Previous studies in Greek breast and/or ovarian cancer patients with family history have shown that four specific *BRCA1* mutations, c.5266dupC, G1738R, and two large genomic rearrangements involving deletions of exons 20 and 24, have a prominent function in the population's *BRCA1* and *BRCA2* mutation spectrum.

**Methods:**

To estimate the frequency of the above mutations in unselected Greek breast cancer women, we screened 987 unselected cases independently of their family history, collected from major Greek hospitals.

RESULTS: Of the 987 patients, 26 (2.6%) were found to carry one of the above mutations in the *BRCA1* gene: 13 carried the c.5266dupC mutation (1.3%), 6 carried the exon 24 deletion (0.6%), 3 carried the exon 20 deletion (0.3%), and 4 carried the G1738R mutation (0.4%). Among 140 patients with early-onset breast cancer (<40 years), 14 carried one of the four mutations (10.0%).

**Conclusion:**

These results suggest that a low-cost genetic screening for only the four prominent *BRCA1* mutations may be advisable to all early-onset breast cancer patients of Greek origin.

Breast cancer is the most common malignancy in women, affecting approximately 1 in 10 women. Incidence rates are increasing annually and it is estimated that by year 2010 there will be 1.5 million new cases worldwide (Parkin *et al*, 2005). Germ-line mutations in predisposing genes, mainly *BRCA1* (MIM 113705) and *BRCA2* (MIM 600185) but also other genes critical for genome integrity such as *CHEK2*, *PALB2*, *ATM*, *p53*, *PTEN*, *STK11*, *CDH1* and more, are responsible for about half of familial breast cancer cases, which in turn represent one-third of all breast cancers (Walsh and King, 2007). Mutations are often found in families burdened with many cases of breast and/or ovarian cancer, usually occurring at an earlier age. Assessing an at-risk individual's mutation status is a procedure growing in clinical importance from an optional genetic test to a powerful predictive tool, as prevention protocols for carriers become established and new treatment options emerge (Robson and Offit, 2007; De Greve *et al*, 2008). However, full *BRCA1* and *BRCA2* screening still remains a tedious task due to gene length, the plethora of mutations, the high frequency of *BRCA1* rearrangements requiring special technical approach, and mutation spectrum differences in different populations. This procedure remains too expensive to target a broader population part and cannot be routinely applied in less privileged areas and countries.

Several studies have shown that in certain countries and ethnic communities the *BRCA1* and *BRCA2* mutation spectrum is limited to a few founder mutations (Neuhausen, 2000; Fackenthal and Olopade, 2007; Ferla *et al*, 2007), a fact that allows more cost-effective screening of individuals with low mutation probability. Testing could thus be made accessible to more women. From such broader studies, it has emerged that many women found to carry a mutation do not have a documented previous family history of the disease.

We have shown in our previous work on the *BRCA1* and *BRCA2* mutation spectrum of the Greek population that four *BRCA1* mutations account for 54% of all mutations detected in both genes, whereas the rest are unique or low-frequency mutations, reflecting the population's genetic heterogeneity (Konstantopoulou *et al*, 2008). As our previous studies involved individuals with medium to strong family history, our aim here is to expand the population screened to breast cancer patients independently of their family history of breast or ovarian cancer. A hospital-based cohort of 987 unselected breast cancer cases was analysed to determine the frequency of the four most common mutations of the *BRCA1* gene. In that way, a population-specific, cost-effective, mini protocol for genetic testing of hereditary breast cancer is applied to a wider population setting and is evaluated for clinical usefulness. As an early age at breast cancer onset is generally considered an indicator of genetic susceptibility, it is interesting to assess whether and in what degree mutation frequencies differ between unselected cases and cases with disease onset before age 40.

## Materials and methods

### Patients

Screening for the four *BRCA1* mutations was performed in 987 unselected female breast cancer patients with histologically verified diagnosis, regardless of family history. The samples were collected from several major hospitals in collaboration with the Hellenic Cooperative Oncology Group (HECOG), representing major geographical areas of Greece, such as Athens metropolitan area, Thessaloniki, Ioannina, Patras, and Crete (Chania). The study was approved by ethics and research committees of the hospitals and was in agreement with the 1975 Helsinki statement, revised in 1983. Informed consent was obtained from all individuals before genetic analysis was performed. The patients' mean age of breast cancer diagnosis was 53.9 years, ranging from 20 to 87 years old. Our cohort included 140 patients with early-onset (<40 years) breast cancer (14.19% of the total), whereas 382 patients (38.66%) were diagnosed before the age of 50. Among the 987 patients tested, 6 have developed bilateral breast cancer (0.6%), 8 have developed both breast and ovarian cancer (0.8%), and 10 have developed both breast and another type of cancer (1.0%). Breast and/or ovarian cancer family history was reported by 111 patients (11%). The minimum criterion to classify a patient as having family history was one additional first- or second-degree relative with breast cancer diagnosed before 50 years or ovarian cancer at any age. Family history was obtained after an interview with each patient. Once a mutation was confirmed in an independent blood sample, results were communicated to patients by the Oncology units participating in the HECOG network. For patients found to be carriers, there were ensuing counselling efforts.

### Mutation detection

Genomic DNA was extracted from peripheral blood lymphocytes using a standard chloroform–isoamyl alcohol protocol. The quantity and the quality of the DNA samples was determined by UV absorbance using a NanoDrop 1000 (Thermo Fisher Scientific, MA, USA) and agarose gel electrophoresis. Detection of the *BRCA1* c.5266dupC (5382insC) mutation was performed using a previously described mutagenically separated polymerase chain reaction (PCR) assay (Chan *et al*, 1999). The *BRCA1* G1738R mutation was detected using an RFLP-based method as previously described in Anagnostopoulos *et al* (2008). To detect the genomic rearrangements involving *BRCA1* exons 20 and 24, we used diagnostic PCR primers as described in Armaou *et al* (2007). In all cases, PCR amplifications were performed in a Veriti 96-Well Thermal Cycler (Applied Biosystems, Foster City, CA, USA). PCR products were electrophoresed through 2.5% agarose gel and visualised by ethidium bromide staining. To confirm each detected mutation, we directly sequenced PCR products using an ABI PRISM Dye Deoxy Terminator Kit v3.1 (Applied Biosystems) in ABI 310 Genetic Analyzer (PerkinElmer, Applied Biosystems), according to manufacturer's instructions. Primer sequences and protocols are available upon request.

## Results

We have screened 987 samples from unselected breast cancer patients for the four most common *BRCA1* mutations in Greece: c.5266dupC (5382insC), G1738R (c.5212G>A), the 3.2 kb deletion of exon 20 (c.5256_5277+3179del3200), and the 4.4 kb deletion of exon 24 (c.5468-285_5592+4019del4429_insCACAG).

**Figure 1:**
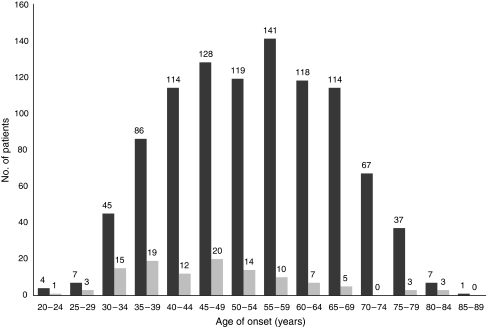
Age of onset distribution among the 987 consecutive breast cancer patients tested. Black bars depict distribution of the total number of patients, whereas grey bars include only patients with family history.

The distribution of the age of onset among all patients studied and among patients with family history is shown in Figure 1. Comparison of the two categories clearly shows a much earlier disease onset in the latter group, with the majority developing breast cancer before 50 years. The mean age of diagnosis for patients with family history was 46.3 years, eight years lower than that for the whole cohort (53.9 years).

Twenty-six patients (2.6, 95% CI 1.6–3.6%) were found to carry one of the four *BRCA1* mutations presented in this study. Mutation c.5266dupC (5382insC) was detected in 13 breast cancer patients (1.3, 95% CI 0.6–2.0%), the exon 24 deletion in 6 patients (0.6, 95% CI 0.1–1.1%), the Greek founder G1738R in 4 patients (0.4, 95% CI 0.01–08%), and the exon 20 deletion in 3 patients (0.3, 95% CI 0.0–0.8%). Table 1 summarises disease and family history characteristics of the 26 mutation carriers.

Among carriers, the mean age of diagnosis was 42.5 years (range 28–61 years), more than a decade lower than the 53.9 years mean age of onset for the whole cohort. Furthermore, 54% of the carriers (14 out of 26) belonged to the ‘early-onset’ group (<40 years) compared to 14% of the whole cohort. Breast and/or ovarian cancer family history was reported by 17 out of 26 carriers (65.4%) compared to 11% of all consecutive breast cancer patients screened.

It is noteworthy that 10% (95% CI 5.0–15.0%) of all early-onset patients carry one of the four *BRCA1* mutations screened, a fourfold increase from the frequency corresponding to the whole cohort (2.6%).

## Discussion

In the present study, we have identified 26 carriers of a *BRCA1* germ-line mutation among a hospital-based series of 987 breast cancer patients unselected for family history, specifically screened for the four most common *BRCA1* mutations in the Greek population. This is the first report of an effort to provide a reliable frequency of those mutations in Greek breast cancer patients, regarding unselected cases as well as early-onset (below 40 years) cases.

Most population studies include cases selected for strong family history of cancer. These have estimated the prevalence of *BRCA1* and *BRCA2* mutations in such high-risk families from 20 to 67% ((Shih *et al*, 2000; Fackenthal and Olopade, 2007; Palma *et al*, 2008), and numerous others), where variation is mainly due to study design and population genetic pool, that is founder populations tend to present higher frequencies. There are also numerous studies with populations selected for age of onset. The frequency of *BRCA1* and *BRCA2* mutations in these studies again varies according to study design (cohort, age that is considered ‘early onset’, mutation screening techniques) and population. For ages of onset between 35 and 45 years and for Western populations with no strong founder effects, mutation frequency has been estimated from 6 to 13% for both genes (Peto *et al*, 1999; Anglian Breast Cancer Study Group, 2000; Malone *et al*, 2000; Syrjakoski *et al*, 2000; Loman *et al*, 2001; de Sanjose *et al*, 2003; Bonadona *et al*, 2005; Lubinski *et al*, 2006). Our findings of a prevalence of 10% for the four common *BRCA1* mutations in Greece in early-onset cases (<40 years) are broadly consistent with the estimates from the previous studies.

Less is known about the prevalence of *BRCA* mutations in the general population or even in unselected breast cancer cases. Most available data originate from founder populations only screened for their founder mutations (Abeliovich *et al*, 1997; Thorlacius *et al*, 1998; Warner *et al*, 1999). The limited number of studies concerning unselected cases in populations with genetic heterogeneity report a frequency of 1.8–4.7% for both genes (for a review, see Fackenthal and Olopade (2007)). For *BRCA1* only, a mutation frequency of 0.4% is found in Finnish (Syrjakoski *et al*, 2000), 2.1% in Dutch (Papelard *et al*, 2000), 3% in Polish (Gorski *et al*, 2005), <1% in Swedish (Margolin *et al*, 2004), 1.5% in Brazilian (Gomes *et al*, 2007), and 2.4–3.5% in US unselected breast cancer patients (Newman *et al*, 1998; Malone *et al*, 2006; John *et al*, 2007). In our present study, we report a total frequency of 2.6% for the four *BRCA1* mutations screened. This result is the minimum estimate and most likely an underestimate of the true frequency of all *BRCA1* mutations in Greek breast cancer patients. The Greek *BRCA1* and *BRCA2* mutation spectrum, as emerged from full screening in high-risk patients, consists of a total of 26 different mutations, 15 in *BRCA1* and 11 in *BRCA2*. Of these, only four *BRCA1* mutations are found in 54% of the families carrying either a *BRCA1* or a *BRCA2* mutation and in 73% of the families carrying a *BRCA1* mutation (Konstantopoulou *et al*, 2008). An extrapolation of the present results in an effort to deduce the true frequency of all *BRCA1* and *BRCA2* mutations in unselected breast cancer patients would suggest that about 4.8% of all Greek breast cancer patients can be carriers of a *BRCA1* or *BRCA2* mutation, as the four mutations screened here (2.6% frequency) only represent 54% of the *BRCA1*/*2*-positive high-risk families. In the same line, 18% of all early-onset patients (<40 years) can carry either a *BRCA1* or a *BRCA2* mutation, given the frequency of 10% found here for the four common *BRCA1* mutations screened. Of the 111 breast cancer patients among our cohort that reported a family history of breast and/or ovarian cancer, 17 have been found to carry one of the four *BRCA1* mutations (15.3%). The lower frequency than the previously reported of 20% can be explained by our use of less strict criteria to define family history and because screening was limited to the four common mutations.

The two categories in our cohort – that of patients reporting family history and that with early-onset (<40 years) of the disease – are interdependent, that is more than one-third (42 out of 111, 38%) of patients with family history are also early-onset cases (see also Figure 1); vice versa, one-third of early-onset cases (42 out of 140, 30%) have family history. Of the 42 patients belonging to both groups, 11 carried a mutation (26%). Table 2 summarises mutation frequencies for the various categories among our cohort.

Half of the carriers identified in the present study (13 out of 26) have *BRCA1* mutation c.5526dup (5382insC). According to previously published results, this mutation accounts for 31% of the *BRCA1* and *BRCA2* mutations identified in Greek familial breast/ovarian cancer patients (Ladopoulou *et al*, 2002; Konstantopoulou *et al*, 2008). As haplotype analysis has shown, *BRCA1* c.5526dup (5382insC) is a ‘founder’ mutation that originated from the Baltic area and has spread worldwide, but is more prevalent in eastern European and Ashkenazi populations (Neuhausen *et al*, 1996; Szabo and King, 1997; Hamel *et al*, 2006; da Costa *et al*, 2008). The frequency of the mutation revealed here (1.3%) is consistent with previous studies performed also in unselected breast cancer patients. A frequency of 1% is reported in Germany (Backe *et al*, 1999), of 1.3% in Brazil (Gomes *et al*, 2007), of 2.1% in Poland (Gorski *et al*, 2005), of 3.7–4% in Russia (Sokolenko *et al*, 2006; Bermisheva *et al*, 2008), and of 2.5–3.8% for Ashkenazim living in the United States (King *et al*, 2003) and Canada (Satagopan *et al*, 2001). Contrary to the case of c.5526dupC, the other three *BRCA1* mutations screened here have been found only in Greek patients.

The present findings have important implications for determining the policy for *BRCA1* and *BRCA2* mutation testing in the Greek population and populations with similar genetic background. It is currently widely accepted that women who fulfil certain criteria based on family history alone or in combination with age of onset should be directed towards *BRCA1* and *BRCA2* genetic testing. The complexities of the particular test prohibit its recommendation to wider targets, such as all breast cancer patients or even the whole population. However, many mutation carriers who do not fulfil screening criteria have been identified before. We have discovered among our cohort six mutation carriers with no reported family history and with later onset of the disease (Table 2). These cases would have been missed if commonly applied (e.g. two cases of breast cancer before age 50) screening criteria were used. As *BRCA1* mutations are highly penetrant and *de novo* formation is extremely rare, the following factors should be considered as an explanation and taken into account when evaluating patients for referral to genetic testing: misreported family history due to lack of knowledge or unwillingness to share information, limited family structure, greater prevalence of male family members, paternal inheritance, also for Greece the lack of a National Cancer Registry. To address this matter, we believe that the simplified *BRCA1* screening for the four most prevalent Greek mutations designed and employed here should be offered to more women with breast cancer, especially those with an early disease onset and ideally to all breast cancer patients. Furthermore, we propose that all early-onset patients should be directed towards full *BRCA1* and *BRCA2* screening as the true frequency of mutations in this group as indicated by our results could be close to 20%.

In conclusion, our results indicate that 2.6% of the Greek patients developing breast cancer independently of age or family history and 10% of Greek patients developing breast cancer before age 40 carry one of four common Greek *BRCA1* mutations. This finding shows the necessity of a new simplified and focused genetic screening approach reaching to a wider population target. Our results also imply that recommendation of full screening of the *BRCA1* and *BRCA2* genes, and even additional predisposing genes, to all breast cancer patients should remain the ultimate goal as new technologies emerge that can lower the cost and effort involved. This point gains additional weight under the view of new personalised therapeutics evolving in the field of oncology.

## Figures and Tables

**Table 1:** Disease history of the 26 breast cancer patients found to carry a *BRCA1* germ-line mutation

**Mutation**	**Patient no.**	**Age of onset**	**Family history of breast and ovarian cancer (age of onset)**	**Comments/other cancers**
c.5266dupC (5382insC)	363	34	Two cousins (p) BrCa52	Father CRC74, sister CRC, uncle (p) stomach ca50
	364	33	Sister BrCa37	
	365	33	Aunt (p) OvCa53, Aunt (p) OvCa54	
	366	35	Mother OvCa45	
	368	39	NO	
	369	36	Mother OvCa, aunt (p) BrCa	Grandmother (m) CRC or stomach ca
	370	58	Sister BrCa and OvCa	Mother gynaecological ca (details not known), father haematological ca
	371	31	Aunt (m) BrCa or OvCa	
	387	37	Sister BrCa32, cousin (m) BrCa	Father pancreatic ca
	412	57	No	
	454	40	Mother BrCa68, mother's sister BrCa52, mother's sister BrCa50, sister BrCa42	
	552	49	No	
	578	36	No	
Exon 24 deletion^a^	284	52	Proband OvCa52, mother's sister BrCa	
	332	28	Proband OvCa31	Father Hodgkin's53, grandmother (p) CRC, grandfather (m) ca urinary bladder, mother's brother Hodgkin's78
	382	29	Mother BrCa32 and BrCa42	Father brain ca55
	440	69	Proband OvCa48	
	442	39	Mother OvCa52, cousin (m) BrCa50, mother's sister BrCa	Grandmother (m) uterine ca, aunt (m) uterine ca52, cousin (m) uterine ca52
	468	39	No	
G1738R^b^	373	47	Sister BrCa27, father's sister bilateral BrCa <50, father's sister BrCa47, father's sister BrCa50	Proband previously had BrCa39, father CRC74
	374	61	No	
	375	57	No	
	466	46	Mother's sister BrCa 47	
Exon 20 deletion^a^	351	43	No	
	428	58	Proband OvCa56, cousin (p) BrCa	Mother CRC, father liver ca, cousins (p) gynaecological ca (details not known), cousin (p) lung ca, father's brother ca larynx, father's sister ca unknown primary
	441	51	No	

Abbreviations: ca, cancer; (m), maternal side; (p), paternal side; BrCa, breast cancer; OvCa, ovarian cancer; CRC, colorectal cancer; number after condition indicates age of onset (where known).

a Described in Armaou *et al* (2007).

b Greek founder mutation (Anagnostopoulos *et al*, 2008).

**Table 2:** Representation and carrier frequency of various breast cancer patient categories in our screening cohort

**Patient category**	**% in cohort (*n*=987)**	**% of carriers in each category/95% CI**	**% of 5382insC carriers in each category**	**% in carriers (*n*=26)**
Total	100% (*n*=987)	2.6 (26/987)/1.6–3.6%	1.3 (13/987)	100 (*n*=26)
Family history^a^	11 (*n*=111)	15.3 (17/111)/8.6–22.0%	8.1 (9/111)	65.4 (*n*=17)
Early onset (<40 years)	14.19 (*n*=140)	10 (14/140)/5.0–15.0%	7.1 (10/140)	54 (*n*=14)
Family history and early-onset (<40 years)	4.25 (*n*=42)	26.2 (11/42)/12.9–39.5%	19 (8/42)	42.3 (*n*=11)
Ovarian cancer^b^	2.13 (*n*=21)	47.6 (10/21)/26.3–69.0%	23.8 (5/21)	38.5 (*n*=10)
No family history, onset >41 years	79 (*n*=781)	0.77 (6/781)/0.2–1.4%	0.26 (2/781)	23.1 (*n*=6)

a By criteria stated in text.

b Personal or family history.
